# An Adapted Cognitive Behavioral Stress and Self-management Intervention for Sexual Minority Men Living With HIV and Cancer Using the SmartManage eHealth Platform: Protocol and Study Design

**DOI:** 10.2196/37822

**Published:** 2022-07-18

**Authors:** Marc Puccinelli, Julia Seay, Amy Otto, Sofia Garcia, Tracy E Crane, Roberto M Benzo, Natasha Solle, Brian Mustanski, Nipun Merchant, Steven A Safren, Frank J Penedo

**Affiliations:** 1 Department of Psychology University of Miami Miami, FL United States; 2 Naval Health Research Center San Diego, CA United States; 3 Department of Public Health Sciences University of Miami Miami, FL United States; 4 Sylvester Comprehensive Cancer Center University of Miami Miami, FL United States; 5 Department of Medical Social Sciences Feinberg School of Medicine Northwestern University Chicago, IL United States; 6 Department of Medicine University of Miami Miami, FL United States; 7 Feinberg School of Medicine Northwestern University chicago, IL United States; 8 Department of Psychology University of Miami Coral Gables, FL United States

**Keywords:** intervention, HIV, cancer, participant, SmartManage, cognitive behavioral stress and self-management

## Abstract

**Background:**

Sexual minority men are disproportionately affected by HIV. Medical advances in HIV treatment have extended life expectancy, and as this group ages, medical and psychological challenges become more prominent. Older people with HIV experience a higher incidence of cancer and other comorbidities; these burdens along with sexual minority stress can strain coping resources and diminish health-related quality of life. Interventions such as cognitive behavioral stress and self-management (CBSM) can mitigate some of this burden; however, no manualized, eHealth-based interventions have focused on the unique needs of sexual minority men living with HIV and cancer.

**Objective:**

This study aims to refine and finalize a web-based, CBSM-based intervention to meet the unique needs of this population, including sexual health, comanagement of 2 chronic conditions, and coping with sexual minority stress.

**Methods:**

This mixed methods study used a previously completed qualitative phase (n=6) to inform the development of a web-based platform and intervention called SmartManage. The pilot phase study (n=50) involved randomization (1:1) into either 10 sessions of adapted CBSM or an attention control health promotion. Both conditions used the SmartManage platform, a web-based eHealth program designed to deliver CBSM and health promotion content and host *live* groups. Feasibility and acceptability (eg, rates of participant engagement and retention) were the primary outcomes.

**Results:**

Participant-related activities are expected to be completed by November 2022, and results are expected to be submitted for publication by February 2023.

**Conclusions:**

We hypothesize that participants would find the intervention acceptable (compared with engagement and retention rates observed in similar CBSM studies). We also hypothesize that participants receiving the SmartManage intervention would have reduced symptom burden and improved health-related quality of life before and after treatment compared with those who do not.

**International Registered Report Identifier (IRRID):**

DERR1-10.2196/37822

## Introduction

With the advent of highly active antiretroviral treatment in the late 1990s, the life expectancy of people living with HIV now nearly matches that of people without HIV. However, the aging cohort of people living with HIV faces substantial health disparities, including higher rates of non–AIDS-defining cancers including liver, lung, and colorectal cancers [1], and for some of these people, these cancers may develop at a younger age [2]. Sexual minority men (SMM) are disproportionately affected by HIV, and many face societal stigma related to both HIV and sexual minority status. The demands of simultaneously managing 2 chronic conditions in the context of this stigma can negatively impact health-related quality of life (HRQoL) and health outcomes. For example, many sexual minority patients miss or delay the needed medical care because of provider bias [3-5]. Relative to heterosexual men, gay men may not be screened for less common cancers in heterosexual communities, such as anal cancer [6,7]. Gay men also report lower satisfaction with their cancer care, which is associated with greater anxiety and poor quality of life [8,9]. Collectively, these challenges place patients who are SMM at a greater risk of late-presenting advanced cancer, leading to worse treatment outcomes and HRQoL.

Psychosocial interventions, including cognitive behavioral stress and self-management (CBSM), can ameliorate symptom burden and improve HRQoL among patients with prostate or breast cancer [10,11]. CBSM also shows favorable effects in SMM (eg, reduced distress and improved mood) [12,13]. However, there are limitations to the standard CBSM for those with HIV and cancer, particularly SMM with HIV and cancer. They face the additional burden of managing complex medical regimens in an often-fragmented care model and in the presence of enduring stigmas around HIV and sexual minority status. Many SMM report experiences with chronic discrimination and nonaffirming providers, leading to apprehension about self-disclosure of sexual minority status to cancer providers or support services (eg, support groups) [14,15]. For group therapy participants and medical patients, societal pressure to conceal their identity can interfere with treatment and leave important needs unaddressed. An equally important challenge is the lack of focus on specific problems for this population, including sexual health and difficulties specific to HIV and cancer comanagement [16].

To address this treatment gap, we are adapting and piloting our CBSM intervention using the *SmartManage* platform (an eHealth-based program for stress management and relaxation training management). SmartManage is a web-based, synchronous platform that hosts all components of our CBSM intervention, which delivers evidence-based techniques to improve self-management, psychosocial or physiological adaptation, and HRQoL. The 9 distinct CBSM intervention targets are listed in Textbox 1.

**Additional treatment targets integrated into adapted cognitive behavioral stress and self-management (CBSM) for issues relevant to sexual minority men (SMM) living with HIV and cancer.**

**Treatment issues**

- HIV and cancer stigma
- Coping with social and medical challenges of 2 major chronic illnesses
- Sexual minority status often disclosed with HIV serostatus
- Medication and treatment adherence for 2 major chronic illnesses
- Care coordination across medical appointments and providers
- Sexual health and intimacy for SMM in the context of cancer and HIV treatments
- Managing treatment fatigue
- Finding appropriate lesbian, gay, bisexual, transgender, and questioning resources for medical information and support and mental health care
- Recognizing and managing cognitive, emotional, and physical effects of minority stress

This initial trial will use CBSM via the SmartManage platform to address the unique needs of SMM who are HIV positive cancer survivors, both related to specific medical concerns and psychosocial factors that contribute to health disparities. Our primary aim is to evaluate the usability, acceptability, and feasibility of the adapted CBSM intervention; our secondary aim is to evaluate the intended effects (eg, improvements in HRQoL and stress management) relative to an attention control condition. In this paper, we describe (1) the intervention development strategy by using both qualitative and quantitative assessments and (2) a single-site randomized controlled pilot study of the adapted CBSM.

## Methods

### Ethics Approval

All study procedures and assessment materials have been approved by the institutional review board at the University of Miami (IRB #20190762) and by the University of Miami Sylvester Comprehensive Cancer Center Protocol Review and Monitoring Committee.

### Study Design

This pilot intervention development study will test the feasibility, acceptability, and intended effects of an adapted CBSM intervention. Pre–pilot testing and qualitative feedback (see Textbox 2 for qualitative interview questions) were used to refine the intervention, which will be tested in a randomized controlled pilot trial.

**Categorical breakdown of qualitative questions for study phase 1 (usability).**

**Aesthetic appearance**

- “What was your first impression of the website?”
- “What words would you use to describe the appearance of the website? Feel free to comment on the following: layout, colors, size, fonts, etc.”

**User experience**

- “In general, how did you feel about using the website?”
- “Was it fairly intuitive to use?”
- “How easily could you locate what you were looking for?”
- “How was the speed of the site? (Ex: did text, images, sound, and/or video take a short or long time to load?)”
- “What did you like about using the website?”
- “What would you change about the website?”
- “What type of device would you be most likely to use for accessing this site: computer, tablet, or smartphone?”

**Content related to health and health care**

- “In general, how much did you like the information presented on the website?”
- “Were the topics we included relevant to your experience as an HIV+ cancer survivor?”
- “What specifically did you learn that you can use to improve your health and well-being?”
- “What problems or challenges do you have that were not addressed appropriately or to your satisfaction?”
- “What else would you like to see included on the site?”

All study procedures involving participants are intended to be completed remotely via secure Health Insurance Portability and Accountability Act–compliant video conferencing software. This includes the documentation of informed consent and administration of assessments, both of which will be captured using the secure REDCap (Research Electronic Data Capture; Vanderbilt University) platform.

**Figure 1:**
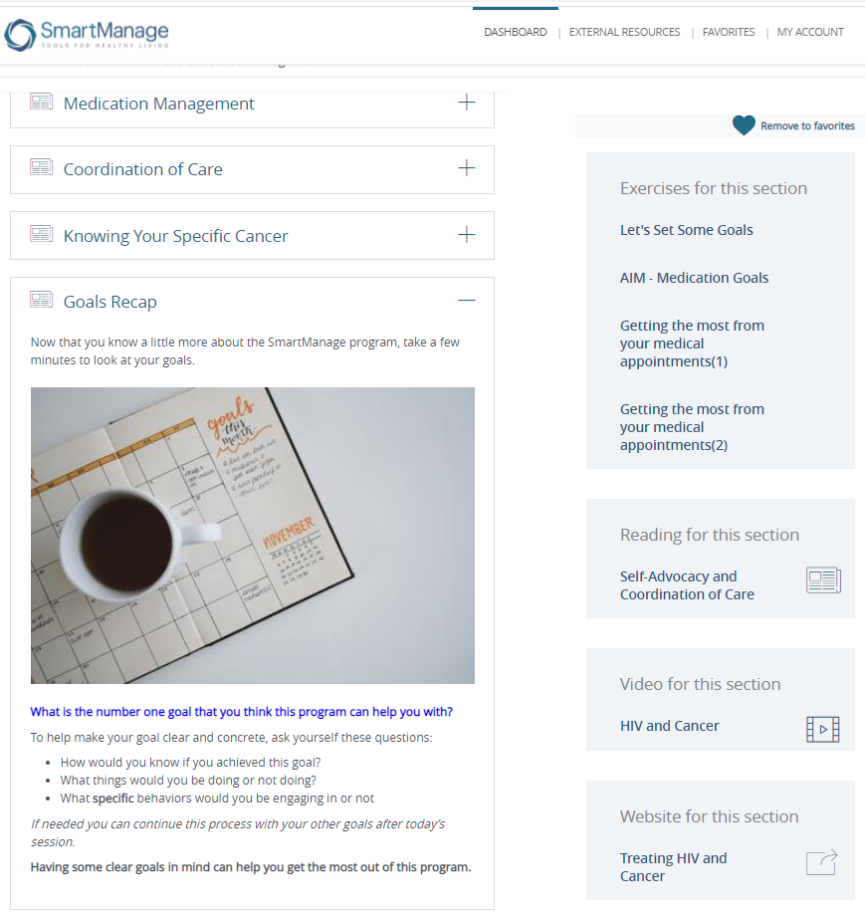
SmartManage session 1 screenshot.

The pilot study will enroll 50 SMM who are HIV positive with a history of a nonmetastatic solid tumor or blood cancer, who will be randomly assigned (1:1) to receive either 10 individual sessions of video-adapted health promotion (HP) content (educational control) or 10 group-based sessions of the SmartManage intervention facilitated by trained interventionists (Figure 1). Before randomization, participants will complete a self-report assessment battery (baseline or T1; Table 1), which they will complete again immediately after treatment (T2). Participants will be compensated US $20 for completing the T1 questionnaire, US $10 for each of the 10 intervention or control sessions, and US $30 for the T2 questionnaire, yielding a maximum of US $150 compensation for completing the study procedures.

**Table 1:** Usability and pilot phase measures.

Measure name	Usability phase	Pilot phase
Demographics Survey (baseline only)	✓	✓
Disease Information Form (baseline only)	✓	✓
ACTG^a^ HIV medication Adherence measure	✓	✓
Barriers to HIV Care survey	✓	✓
Participant Survey on Website Feedback (ie, USE^b^ Questionnaire; postintervention)	✓	✓
Coping Self-Efficacy Scale		✓
Interpersonal Support Evaluation List		✓
Bidimensional Acculturation Scale for Hispanics		✓
Computer Proficiency Questionnaire		✓
Perceived Stress Scale (General Stress)		✓
Impact of Event Scale (disease or dual diagnosis–related distress)		✓
Functional Assessment of Cancer Therapy Scale-General (HRQoL^c^)		✓
Medical Outcomes Study HIV Survey (HRQoL)		✓
SmartManage Acceptability Evaluation (postintervention only)	✓	✓
Communication Assessment Tool-revised		✓
International Physical Activity Questionnaire—short form		✓
PROMIS^d^ Bother Regarding Sexual Function		✓
PROMIS Factors Interfering with Sexual Satisfaction		✓
Pittsburgh Sleep Quality Index		✓
COVID-19: Impact of the pandemic and HRQoL on patients with cancer and survivors		✓
COVID-19: Impact on sexual health—SGM^e^		✓
Everyday Discrimination—Sexual Orientation		✓

^a^ACTG: AIDS Clinical Trials Group.

^b^USE: Usefulness, Satisfaction, and Ease of Use.

^c^HRQoL: health-related quality of life.

^d^PROMIS: Patient-Reported Outcomes Measurement Information System.

^e^SGM: sexual and gender minority.

Participants in phase 1 and participants who are randomized to the CBSM SmartManage intervention (Table 2) condition in phase 2 will be asked to consent to audio recordings of these visits for qualitative analysis and fidelity monitoring. The weekly topics of the educational control HP condition are listed in Textbox 3.

**Table 2:** SmartManage cognitive behavioral stress and self-management for HIV and cancer weekly session topics and key subtopics.

Weekly sessions	Major topic	Subtopic	Relaxation exercises
Session 1	Introduction: HIV and Cancer Comanagement	Goal setting	—^a^
Session 2	Stress and Stress Management	Medication adherence	Breathing meditation
Session 3	Linking Thoughts and Emotions	Cognitive appraisal	Introduction to meditation and mindfulness
Session 4	Linking Thoughts and Emotions: Part 2	Cognitive distortions	Meditation for working with difficulties
Session 5	Sex and Intimacy	Focus on SMM^b^ sexual health	Loving kindness meditation
Session 6	Effective Communication and Managing Emotions	Anger management	Body scan for sleep
Session 7	Partnering With My Health System or Providers	Assertiveness training	Breathing meditation (audio)
Session 8	Social Connections	Building a social support network	Sitting meditation
Session 9	Healthy Behaviors	Coping with information overload	Brief “stop” meditation
Session 10	Wrap up and Program Summary	Plan for continuing work toward goals	Brief 3-minute meditation

^a^None.

^b^SMM: sexual minority men.

**SmartManage educational control health promotion topics.**

**Weekly sessions and topic**

Session 1: Introduction to health promotion

Session 2: Disease and healthy lifestyle

Session 3: Aging

Session 4: Exercise

Session 5: Diet

Session 6: Memory and cognition

Session 7: Sexuality

Session 8: Quality of life

Session 9: Information overload

Session 10: Review and summary

Textbox 3 describes weekly educational control sessions.

### Participants

We enrolled 6 qualitative participants (phase 1) and will enroll 50 pilot participants (phase 2) recruited from specific departments within the University of Miami Hospital (Infectious Diseases and AIDS Clinical Research Unit), University of Miami medical affiliates (Sylvester Comprehensive Cancer Center), consent to contact databases (Center for HIV and Research in Mental Health—CHARM Registry), and via social media.

Participants will meet the following inclusion criteria: (1) ≥18 years of age; (2) fluent in English; (3) diagnosed with at least 1 form of nonmetastatic solid tumor or blood cancer; (4) ≥30 days after the completion of active primary treatment (eg, surgery, radiation, and chemotherapy); (5) self-identify as a sexual minority, cisgender man (ie, self-identify as something other than heterosexual or straight; assigned male at birth and identify as male); (6) self-report having been diagnosed with HIV; and (7) have reliable access to a device with internet access. Adjuvant therapies such as hormone treatment for prostate cancer are not considered exclusionary. Participants will be excluded if they (1) have one of the following exclusionary cancer types: nonmelanoma skin cancer only, brain cancer, eye cancer, or remote history of pediatric cancer only without a history of cancer as an adult; (2) have a history of metastatic cancer of any type; (3) are currently undergoing primary treatment for their cancer; (4) have had inpatient treatment for serious mental illness in the past 12 months, overt signs of serious mental illness, or moderate or higher risk of suicidality at the time of screening; (5) appear actively intoxicated or otherwise unable to provide full informed consent; or (6) have any medical conditions resulting in a predicted life expectancy of <12 months per participant self-report. The intention behind the specificity of these inclusion and exclusion criteria is to minimize the enrollment of participants whose illness is likely to significantly interfere with their ability to engage with the study material (eg, eye cancer) or complete study participation (eg, metastatic illness with a life expectancy of <12 months). The research team felt that this was necessary given the small sample size of this study; however, we also recognize that some patients excluded from this study could benefit from the intervention. We intended to expand inclusion criteria in larger subsequent studies to allow broader medical inclusion and applicability to participants of other genders and sexual orientations.

### SmartManage for HIV and Cancer Survivors: Adapted CBSM for Lesbian, Gay, Bisexual, Transgender, and Questioning Individuals Dually Diagnosed With HIV and Cancer

**Figure 2:**
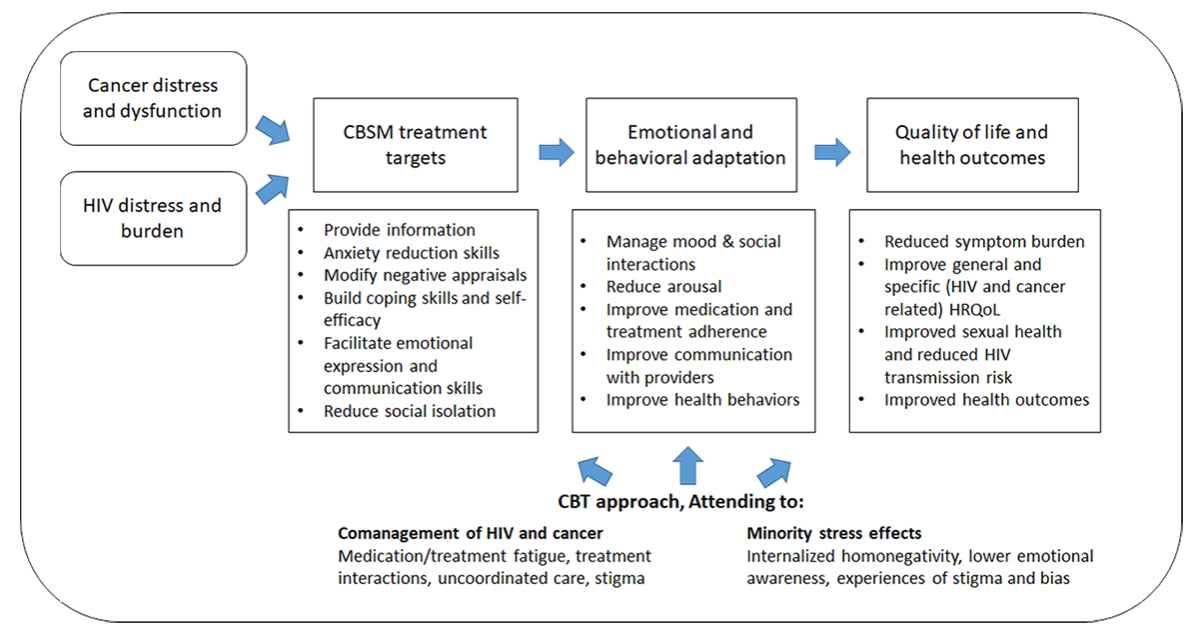
Integrated model of cognitive behavioral stress and self-management (CBSM) for comanagement of cancer and HIV. CBT: cognitive behavior therapy; HRQoL: health-related quality of life.

The SmartManage CBSM intervention was adapted from the manualized CBSM intervention [17], with an additional focus on psychosocial issues relevant to SMM, as well as focus on comanaging HIV and cancer. The adapted CBSM SmartManage for HIV and cancer intervention incorporates cognitive behavioral therapy techniques to facilitate changes in domains known to impact symptom burden, HRQoL, physical functioning, and self-management (see Table 2 for the session outline). The CBSM intervention using the SmartManage platform seeks to promote coping and resilience through the use of practical tools (relaxation training, increasing physical activity, and social support), general and diagnosis-specific information, and cognitive behavioral therapy–based strategies (eg, cognitive restructuring), with added focus on the role of sexual minority stress and issues relevant to SMM (see Figure 2 for conceptual model). Throughout the 10 sessions that lasted for 90 minutes, participants are taught to recognize the antecedents of negative mood and systematically evaluate cognitive distortions to improve symptom management, interpersonal adjustment, and HRQoL. Intervention groups consist of 4 to 6 participants led by a trained therapist (master’s level or psychology doctoral students) with experience in working with sexual minority clients. Participants are encouraged to share relevant experiences and to practice skills during the session. For example, a participant who expresses anger that a friend did not call him back may apply Socratic questioning with the help of the group to identify and challenge cognitive distortions. Participants will be asked to complete live worksheets through the web-based platform, and they will be asked to complete weekly home exercises using downloadable materials. Therapists will troubleshoot and track the prior week’s home practice at the beginning of each subsequent session.

To adapt the intervention for SMM comanaging HIV and cancer, we drew upon our earlier work and newer research with sexual and gender minority clients. Our prior research on interventions to manage prostate cancer survivorship or HIV infection in SMM guided content changes that may be important for SMM with HIV and cancer. We also conducted qualitative interviews with this cohort to determine the usability of the SmartManage website and to allow participants to share what they felt was lacking in the nonadapted interventions [10,18]. We then incorporated these changes within the conceptual framework of the minority stress model, which posits that chronic exposure to societal stigma and bias drives internal stress processes that exacerbate psychological distress and increase the risk of maladaptive coping strategies [19,20]. The resulting CBSM intervention for HIV and cancer using the SmartManage platform therefore includes changes ranging from cosmetic (SMM-specific images and text) to content, with an emphasis on intervention elements most likely to address the interaction of minority stress and the comanagement of cancer and HIV.

### HIV and Cancer SmartManage Content and Format

Participants in phase 2 of the study will be randomly assigned to receive 10 sessions of either the SmartManage CBSM intervention or SmartManage-based HP educational control. Both interventions will be delivered weekly over 10 weeks. Given that this study is intended primarily for intervention development and evaluation of usability, feasibility, and acceptability among the target population, the intervention and control conditions are not matched in terms of dose (session length) or format. Both conditions provide participants with broadly relevant HP information; however, the control content does not contain population-specific adaptations (eg, focus on minority stress) or active psychotherapeutic techniques (eg, cognitive restructuring). Each week involves a prescheduled live web-based group meeting with 4 to 6 members led by a therapist who delivers the session material while collaborating with the participants to guide them through exercises and elicit personally relevant content. Weekly relaxation and stress management topics for the SmartManage-based CBSM condition for the HIV and cancer condition are listed in Table 2. Specific intervention techniques designed to address HIV-, cancer-, and SMM-specific challenges are listed in Textbox 4. Sessions will be audio recorded for later analysis and fidelity rating. Group members will be addressed by their first name only to limit the potential disclosure of personal information. Therapists will discuss with the participants the standard rules and expectations of group treatment, including the importance of maintaining group confidentiality. They will also remind participants of the limits of confidentiality as described in the consent form, acknowledging the possibility that another group member could potentially disclose one’s personal information.

**SmartManage cognitive behavioral stress and self-management for HIV and cancer intervention techniques to address HIV and cancer and sexual and gender minority community–specific challenges. HRQoL: health-related quality of life.**

**Topic**

**Minority Stress Model**

- Introduce model—Acknowledge that minority stress has a measurable negative effect on mental health and HRQoL
- Explore participants’ experiences with stigma and bias
- Validate that the onus for minority stress lies with society and bias, not with the minority group

**HIV and cancer comanagement**

- Explore and problem solve individual and common challenges related to managing cancer and HIV (eg, medication adherence, managing multiple provider appointments, and coordination and communication with providers)
- Screening for secondary cancers, recurrences, and disease progression

**Sex and intimacy**

- Strategies to prevent HIV transmission with serodiscordant partners
- Talking to providers about sexual health concerns, including anal sex
- Problem solving and practical skills to manage sexual difficulties related to illness (HIV or cancer), treatment side effects, and aging

**Interpersonal**

- Acknowledge that internalized homonegativity is often related to less assertive behavior
- Assertiveness skill building

### HP Educational Control

We adapted a manualized, didactic HP workbook to be delivered to the control participants using the SmartManage web-based format. The content for each of the 10 sessions was converted to a slide format, which we then converted into videos with voiceover narration. The session duration ranged from 10 to 20 minutes, and topics included descriptions of age-related and other diseases (eg, diabetes and cardiovascular disease), complications that may arise in the course of each condition, information on various disease treatments and side effects, cancer and HIV information, and other HP content. The control material does not include any content or instruction related to stress and self-management techniques or other psychotherapeutic content. Participants will be invited to log onto the web-based platform to view 1 new video every week over the course of 10 weeks. Built-in diagnostic software will track when participants have finished viewing each video. The HP topics are listed in Textbox 3.

### Data Collection

#### Sociodemographic and Medical History or Status

Participants in both phases will report sociodemographic data including age, race, ethnicity, sex assigned at birth, gender identity, education, income, and household makeup. Participants in both phases will also self-report information about their cancer, HIV, and relevant health behaviors. This includes diagnosis dates, past and current treatments, and medication or treatment adherence. Cancer-specific questions include the type of cancer and whether their cancer has metastasized. HIV-specific questions relate to the frequency of contact with HIV care providers, current viral load, and CD4 count. We will also ask about any barriers they face regarding access to HIV care, such as difficulty obtaining medications or feeling stigmatized by health care providers. Participants in phase 2 will be reassessed after the intervention for information that may change (eg, HIV viral load and cancer prognosis). Table 1 lists all study measures collected.

#### Primary Outcomes: Feasibility and Acceptability

As the main purpose of the SmartManage study is intervention development, quantitative primary outcomes are focused on participant engagement in phase 2 (pilot testing). For the adapted SmartManage CBSM for HIV and cancer intervention to be considered feasible, 70% of the participants who begin the intervention will attend at least 70% of all intervention sessions, and 85% of the enrolled participants will be retained throughout the study. These proportions are based on previously successful CBSM trials in other populations [21-23]. We will also examine cost indicators of implementation feasibility by documenting personnel time, space requirements (eg, for intervention delivery and study administration), and supply costs, including the maintenance and management of the SmartManage website.

We will evaluate intervention acceptability in two ways: (1) the proportion of eligible SMM in phase 2 who agree to participate versus decline (≥30%) and (2) via 2 exit surveys administered after participants have completed the SmartManage intervention. One such survey is the commonly used Usefulness, Satisfaction, and Ease of Use (USE) questionnaire [24], which assesses participants’ impressions of the intervention’s usefulness, ease of use, ease of learning, as well as overall satisfaction with the intervention. We also created an intervention-specific measure to evaluate participants’ views on SmartManage elements that are not assessed in the USE questionnaire. These include perceptions of relevance to health needs, how helpful the program is for SMM with HIV and cancer, and the willingness to recommend the program to similar peers. Both intervention acceptability measures ask participants to rate items on a 7-point Likert scale ranging from *strongly agree* to *strongly disagree*. Both measures also include qualitative questions with open text response fields allowing participants to share thoughts on what they found most and least helpful, what important areas were not addressed sufficiently, and recommendations to improve the intervention.

#### Secondary Outcomes: Intended Effects of Intervention, Other Psychosocial Factors, and Nature of Illness

##### Intended Effects of Intervention

This study is not intended to test the efficacy of SmartManage; however, we will examine the intended treatment effects of the intervention. We expect that relative to the educational control condition, SMM who participate in SmartManage-based CBSM for HIV and cancer will show improvements in health behaviors, HRQoL or disease-related distress, stress, and coping. Health behaviors include adherence to HIV medication regimen, as measured by the AIDS Clinical Trials Group HIV medication adherence measure [25], and frequency and duration of exercise, as measured by the International Physical Activity Questionnaire-Short Form [26]. HRQoL is captured by two measures: Functional Assessment of Cancer Therapy Scale–General [27] and Medical Outcomes Study HIV Survey [28]. Both measures assess illness-related physical, social, and functional well-being as influenced by cancer or HIV, respectively. We will also capture the impact of sexual dysfunction by using the Patient-Reported Outcomes Measurement Information System—Bother Regarding Sexual Function scale [29]. The general stress level over the past 30 days will be measured using the Perceived Stress Scale-14 [30]. Finally, aspects of distress and coping will be assessed using two measures: the Impact of Event Scale [31], which asks participants how much they have been bothered over the past week by memories and intrusive thoughts about past stressful events, and the Coping Self-Efficacy Scale [32], which measures the confidence that one can use a wide range of coping strategies when faced with adversity. Table 1 lists the study measures.

##### Other Psychosocial Factors

Although the SmartManage CBSM for HIV and cancer intervention is designed to address common targets of psychotherapy (stress management and coping skills), we will also conduct exploratory analyses to examine more stable and dispositional factors that may moderate intervention effects. Among these are the frequency and emotional impact of chronic social stigma and bias, which will be measured using the Everyday Discrimination—Sexual Orientation instrument [33], modified to include sexual orientation-specific questions. We will also look at the acculturation level using the Bidimensional Acculturation Scale [34], which assesses *Americanism* and *Hispanicism* independently to categorize individuals according to the quadrant model of acculturation by Berry [35]. This is particularly relevant in Miami-Dade County, where >53% of residents are foreign born and nearly 70% identify as Hispanic [36].

##### Nature of Illness

The target population for SmartManage for HIV and cancer is likely to be more heterogeneous than that of prior CBSM studies, given the widely varying impact of different cancer types. Therefore, we will examine the variability in intervention responses based on specific types of cancer (eg, prostate vs lung). We will also explore the practical and psychological impact of COVID-19 using 1 measure specifically for patients with cancer and 1 to assess how COVID-19 has affected the sexual health and behavior of SMM, both developed by Penedo et al (Penedo FJ, unpublished data, May 2020).

### Data Analytic Plan

#### Aim 1: Conduct Usability Testing and Finalize SmartManage for HIV and Cancer Intervention

For phase 1, we will summarize the participants’ demographic, psychosocial, and clinical information by using descriptive statistics. Audio-recorded participant responses will be transcribed, coded, and qualitatively analyzed using NVivo Pro (version 12.6; QSR International) software. Two independent raters will develop a codebook following a conventional content analysis approach in which codes emerge solely from the data. Throughout coding development, raters will use the constant comparative method to identify themes. Coding will follow an iterative process in which each preceding group will refine themes to accommodate new information until saturation is reached and raters reach consensus. The larger study team will review a summary of the findings to inform and refine the development of the program modules, program content, study documents (including recruitment materials), and study procedures.

#### Aim 2: Randomized Controlled Pilot Testing of Intervention (Feasibility, Acceptability, and Intended Effects)

For phase 2, descriptive statistics will be used to characterize the sample and inspect data quality. We will examine the amount, pattern, and randomness of missing data to determine appropriate statistical methods to handle missingness. Type 1 error will be set to 5% (α=.05) for calculating CIs and performing hypothesis testing. The α values will be adjusted for multiple comparisons, as needed.

We will examine feasibility via engagement and retention rates as well as cost indicators. Intervention acceptability will be evaluated using continuous data from the exit survey (USE questionnaire). These analyses will be primarily descriptive; however, we will use general linear modeling (eg, independent samples 2-tailed *t* test and ANOVA) to determine whether there are significant differences in feasibility and acceptability by sociodemographic, medical, and psychosocial variables. We will examine the distributions to determine whether alterations to the data analytic plan are needed, for example, using nonparametric methods. If >10% of the data are missing completely at random, we will use multiple imputation techniques.

To evaluate the intended effects of the intervention, we will analyze continuous scores on measures of stress, disease-related distress, and HRQoL. We will use paired samples *t* tests to examine whether these variables improve significantly within groups from pre- to postintervention measurement. We will use repeated measures ANOVA to determine whether these potential changes remain significant, accounting for the sociodemographic, medical, and psychosocial covariates described earlier. We will also use independent samples *t* tests to analyze potential differences in outcomes between the study groups (SmartManage intervention vs educational control). These will be analyzed using repeated measures ANOVA to explore whether effects remain when accounting for sociodemographic, medical, and psychosocial covariates.

## Results

Participant qualitative enrollment began in February 2022, and phase 2 enrollment began in April 2022. All intervention and assessment procedures are expected to be completed no later than November 2022. Both the qualitative and quantitative outcomes are expected to be submitted for publication by February 2023.

## Discussion

In this study, we seek to build on prior research by incorporating population-specific content and emphasizing targeted areas of treatment to address the disproportionate health burden on SMM living with HIV and cancer. We drew upon relevant theoretical models (minority stress and syndemic theory) that have shown their utility in guiding the development of efficacious interventions for marginalized populations [37,38]. Although the appropriate application of theory and thorough evaluation of prior research are necessary steps in developing an intervention tailored for the sexual and gender minority community, we also recognize the vital contribution of stakeholder feedback and involvement in this process [39]. Therefore, the primary purpose of this study was to systematically evaluate participant feedback and provide insights to guide further refinement of the treatment model. We also anticipate that those who receive the experimental intervention will show measurable improvement in stress burden, coping self-efficacy, and overall HRQoL.

Despite improved HIV treatment that allows those living with the virus to live longer and healthier lives relative to those infected earlier in the HIV epidemic, the large and growing cohort of aging SMM living with HIV continues to face stigma and bias related to HIV status and sexual minority status, as well as unanticipated health problems, including higher rates of cancer. Together, these factors contribute to poorer HRQoL, diminished rates of cancer survivorship, and higher rates of mental health concerns such as depression and anxiety [40,41]. There are few intervention programs intended to address the specific needs of SMM living with HIV and cancer despite the widely acknowledged critical role of mental health treatment in ending the HIV epidemic [42]. We seek to further the process that will eventually address this need by using qualitative and quantitative (mixed) methods to develop an intervention program to address the disproportionate symptom burden and diminished HRQoL among SMM living with HIV and cancer.

The rationale for creating this adapted intervention for a relatively small subset of the total population is similar to the reasons behind specialized interventions such as panic control treatment [43] or any culturally adapted treatment, that is, unmet needs in an identifiable community, and significant negative consequences that could be ameliorated with appropriate care. The need is even more pronounced in our target population (SMM with HIV and cancer) because of the syndemic conditions that both concentrate disease risk and maintain conditions detrimental to the overall health trajectory over time within minority populations [44-48]. This study is not designed to determine whether the disproportionate health burden present in our target population is attributable to syndemic factors; however, we do know that the effects of societal bias and minority stress are ubiquitous within this group. Without intervention, the ongoing corrosive impact of these and other syndemic factors create a high likelihood of suboptimal health trajectories in this population. Therefore, our approach is a person-centered method of providing the necessary tools and skills via an intervention that integrates evidence-based techniques to improve self-management, self-efficacy, communication skills, stress awareness, and management to improve physiological and psychosocial adaptation on the one hand and HRQoL and health outcomes on the other hand. It is a broader goal that social and structural factors that lead to syndemic conditions can be addressed such that negative health outcomes can be prevented. Therefore, our immediate goal is to facilitate improved coping and offer practical strategies that can improve health outcomes in this critically challenged subgroup of cancer survivors.

Although this is a pilot study intended as an early developmental stage for the treatment model, there are some inherent limitations. One limitation is the inclusion of participants who have many types of cancer because of the relative infrequency of study participants with both HIV and cancer. The progression of illness, nature of treatment, and common sequelae of different forms of cancer can vary widely. We also hope to further tailor the interventions to address the specific needs of the participants. Some participants will likely have unique needs that cannot be fully addressed in a time-limited group session format. We anticipate that these data gathered in this study will allow us to refine the intervention to address the most pressing needs and guide the creation of a comprehensive resource where participants can get help with problems not addressed in the session. Wider implementation of this model would allow further refinement and could facilitate more targeted cancer groups (eg, prostate cancer and blood cancer), given that the web-based format allows participants to join from any location.

Similarly, we recognize that limiting enrollment to those with nonmetastatic cancer who have completed primary cancer treatment excludes a significant number of potential participants. Active treatment and metastasis have a high potential to interfere with group attendance, which is problematic given the small sample size of this study. In future iterations, we will seek to broaden the inclusion criteria and tailor the content to address the acute needs of such individuals.

We also recognize that most people in our current catchment area (South Florida) are Hispanic, and this study is conducted only in English. This may limit our ability to generalize feasibility and acceptability findings, given a large number of Spanish monolingual speakers. To address this limitation, our collaborators are culturally adapting the SmartManage intervention for Hispanic participants to be tested concurrently with this study. This culturally adapted version will be piloted in English and then translated and tested in Spanish.

In addition, we believe that a web-based intervention is a significant strength; however, it is also a potential limitation. The intent of this format is to make group sessions accessible to those for whom in-person meetings are not feasible owing to physical limitations, lack of transportation, and location of residency. However, the requirement for a video-equipped device and stable internet is prohibitive for those who are most vulnerable, including those who are unstably housed. A potential solution that we used for other studies during the COVID-19 pandemic is to provide computer-equipped therapy rooms where participants can join the web-based group in a secure, private setting. This requires accessible facilities and reintroduces the travel burden, both of which may require additional creative solutions.

We expect that this study will inform the development of an intervention to specifically address the needs of an underserved and highly burdened population. This study will provide much-needed information regarding the utility and acceptability of a web-based group format, which will inform future iterations, bringing treatment options to those without access. We also expect that the information gathered will contribute to the literature by providing both qualitative and quantitative data describing the experiences and needs of a marginalized population living with multiple chronic illnesses. Although this is a relatively small sample, the lessons learned may be generalizable to other marginalized communities facing multiple challenges and may inform large-scale randomized controlled trials with long-term follow-up to assess the clinical utility of these programs.

## Abbreviations

CBSM: cognitive behavioral stress and self-management
HP: health promotion
HRQoL: health-related quality of life
REDCap: Research Electronic Data Capture
SMM: sexual minority men
USE: Usefulness, Satisfaction, and Ease of Use

## Data Availability

As this is a pilot intervention development study, outcome data will not be publicly available but will be available from the corresponding author (FJP) on reasonable request.
